# Borderline Personality Disorder Symptoms and Its Clinical Correlates among Chinese University Students: A Cross-Sectional Study

**DOI:** 10.3390/healthcare10091751

**Published:** 2022-09-12

**Authors:** Nan Jia, Chaiyun Sakulsriprasert, Nahathai Wongpakaran, Chawisa Suradom, Ronald O’ Donnell

**Affiliations:** 1 Master of Science (Mental Health), Graduate School, Chiang Mai University, Chiang Mai 50200, Thailand; 2 Department of Psychology, Faculty of Humanities, Chiang Mai University, 239, Huay Kaew Road, T. Suthep, A. Muang, Chiang Mai 50200, Thailand; 3 Department of Psychiatry, Faculty of Medicine, Chiang Mai University, 110 Intawaroros Rd., T. Sriphum, A. Muang, Chiang Mai 50200, Thailand; 4 College of Health Solutions, Arizona State University, 500 N. 3rd St., Phoenix, AZ 85004, USA

**Keywords:** borderline personality, university students, depression, attachment, self-esteem, perceived stress, resilience, correlates, China

## Abstract

Borderline personality disorder (BPD) is common among young adults. Related studies showed a wide range of prevalence among university students. Few studies regarding BPD symptoms and their correlations with different variables have been reported in the Chinese population. A cross-sectional, online survey was conducted on a sample of university students in China between November 2021 and January 2022. Sociodemographic questionnaires, the Screening Instrument for Borderline Personality Disorder (SI-Bord), the 18-item Experience in Close Relationships-Revised (ECR-R-18), the Meaning In Life Questionnaire (MLQ), the 10-item Perceived Stress Scale (PSS-10), the Patient Health Questionnaire (PHQ-9), the Rosenberg Self-Esteem Scale (RSES) and the Resilience Inventory (RI-9) were completed. Data were analyzed using Pearson’s correlation methods. Among 767 participants, mean age was 20.33 ± 1.495 years, and the majority were males (53.5%). According to the SI-Bord’s cut-off score >7, BPD symptoms were found in 17.5% of participants. Attachment anxiety, avoidance, depression, perceived stress, lack of meaning in life, resilience and self-esteem were significantly correlated with BPD symptoms with r’s of 0.473, 0.180, 0.451, 0.481, −0.148, −0.238 and −0.388, respectively (all p’s < 0.01). The prevalence of BPD symptoms is high among Chinese university students and significantly associated with mental health outcomes, suggesting that an early detection of BPD symptoms is necessary for this population.

## 1. Introduction

Borderline personality disorder (BPD) usually occurs during adolescence [1]. DSM-5 symptoms of BPD include unstable emotions and interpersonal relationships, identity problems, impulsivity, feelings of emptiness and boredom, as well as self-harm [2,3,4]. It has been found that the suicide rate among people with BPD may be as high as 10%, which is almost 50 times higher than those of the general population [1]. BPD was found to be higher than depressive symptoms in predicting suicidality among Thai university students, (OR 1.19 vs. 1.16) [5]. This finding was in line with a study among Thai adults in which BPD showed a higher predicting effect for suicidal attempts than depressive disorders (OR 8.439 vs. 4.62) [6].

The literature review demonstrated that the prevalence of BPD among university students ranged from 0.5 to 32.1% and varied from time to time, as well as from culture to culture [2,7]. In China, the prevalence of BPD among Chinese undergraduate students ranged between 0.67 and 17.7% [8,9,10,11,12,13,14].

From a developmental perspective, BPD is attributable to insecure attachment pertinent to childhood traumatic experiences [15,16,17,18,19,20], whereas a secure attachment style is a protective factor regarding psychopathological development, as it can protect against deficits in emotional processing and somatization of negative emotions and stressful life [21,22,23]. Individuals with BPD usually express low self-esteem (SE) [24], limited resilience [25] and lack of meaning in life (MIL) [26,27]. BPD is also associated with various mental health outcomes or psychiatric disorders such as perceived stress, depression and suicidality [6,28,29]. Furthermore, one study has demonstrated that suicidality is related to BPD even more than depression among university students [5]. This highlights the important place BPD holds for university students’ mental health.

Despite the fact that the prevalence of BPD has been reported in China for the past ten years, only two papers showed the prevalence of BPD symptoms among university students. In 2013, one of them reported a prevalence of 1.74% and another in 2018 reported an estimated prevalence of 15% [8,9]. During the COVID-19 pandemic, many studies reported increasing mental health problems such as psychological stress, depression, posttraumatic stress disorder [30] and many other factors that could worsen psychological symptoms, such as stress and depression among those with BPD. According to the diathesis-stress theory, people with poor stress management skills are more likely to experience increased stress levels, emotional dysregulation and related maladaptive behaviors. As the COVID-19 crisis persists, people with BPD are vulnerable and tend to experience stress from the ongoing situation [31]. Furthermore, research showed that public health measurements like social distancing and massive indoor self-quarantine could intensify feelings of emptiness and aggravate the fear of abandonment among people with BPD and other distressing emotions [32]. It remains evident that anxiety and depression are tremendously increasing among people during the three years of the COVID-19 pandemic, especially among those who are prone to stress, such as individuals with BPD. 

In addition to BPD, university students might feel stressed due to academic elements, e.g., class competition, and nonacademic burdens, e.g., financial limitations, homesickness, social problems and sleep problems, which leads some to engage in self-destructive behaviors [33,34,35,36]. During an emerging adult life, some of these young people are experiencing existential issues and lack of MIL [37]. Having a sense of MIL has been acknowledged as a catalyst to psychological flourishing. It can raise psychological well-being, improve happiness and life satisfaction and increase interpersonal functioning [38]. Moreover, some other positive attributes such as resilience, a construct representing the maintenance of positive adaptation despite significant adversity [39], represents a growing body of research among university students [40]. 

University students are a country’s hope and superior strength, as China has one of the largest populations in the world. With the prevalence of BPD varying over time, and a strict COVID-19 preventive measures in China, this study aimed to examine the prevalence of BPD symptoms among Chinese university students and its correlations with the aforementioned negative associated factors, i.e., depression and perceived stress, and the positive associated factors, i.e., self-esteem, MIL and resilience outcomes.

## 2. Materials and Methods

This study constituted a cross-sectional online survey between November 2021 and January 2022. The sampling method was purposive, a convenience sampling was used in this study, and a designed Microsoft form questionnaire was used to collect data. Chinese university undergraduate students aged between 18 and 25 years who could understand, speak, read and write in Chinese and have access to an electronic device with an internet connection, e.g., computer, smartphone or tablet, were included in the study. Those who were currently diagnosed with schizophrenia or a bipolar disorder, epilepsy or an organic brain disease, were intoxicated by alcohol or substance abuse within the past 24 h and had active suicidal ideation were excluded. First, we created a poster including the QR code of the Microsoft form questionnaire. Then, we distributed it through the online platform WeChat moments (Version 8.0.20, Tencent, Beijing, China), friends’ circle, QQ (Version 8.9.0.625, Tencent, Beijing, China), TikTok (Version 22.3.0, Douyin Group (HK), Limited, Beijing, China), the websites of www.douban.com and www.sina.com. We also posted the flyer among students’ online networks in different regions of China for 10 universities weekly during the data collection. Students who saw the poster and met the inclusion and exclusion criteria could participate, and they would receive 5 RMB as compensation for their time volunteering in this research.

At the beginning of the questionnaire, the participants’ eligibility information sheet and the informed consent form were used to instruct the participants, and the QR code of the survey was made available on social media. We monitored the link and closed it after meeting the expected number. 

### 2.1. Instruments

#### 2.1.1. Screening Instrument for Borderline Personality Disorder (SI-Bord)

SI-Bord consists of five questions representing the DSM-5 criteria of BPD for abandonment avoidance, interpersonal relationships instability, identity disturbance, suicidal and self-harm behaviors as well as affective instability. The sensitivity is 0.75, and the specificity is 0.73, according to the cutoff score of >7 [28]. The Cronbach’s alpha of the Chinese version tested among 39 Chinese participants between 19 and 29 years old was 0.738 before its use. The Cronbach’s alpha in this sample was 0.724. 

#### 2.1.2. Meaning in Life Questionnaire (MLQ)

The Meaning in Life Questionnaire could measure the presence of meaning in life, as well as the Search for Meaning in Life [41]. The MLQ exhibited excellent internal consistency, test–retest reliability and stable factor structure. The Chinese version displayed good psychometric properties [42]. It takes about 3 to 5 min to complete. In this sample, Cronbach’s alpha was 0.834.

#### 2.1.3. Perceived Stress Scale (PSS-10)

PSS-10 measures the perception of stress. It measures the degree to which situations in one’s life are appraised as stressful [43].The overall Cronbach’s alpha of the Chinese version of PSS-10 was 0.91, and the test–retest reliability was 0.69 [44]. In this sample, Cronbach’s alpha was 0.765.

#### 2.1.4. Resilience Inventory (RI-9)

RI-9 measures the degree of resilience. It captures how much an individual recovers well after a setback or problem [45]. The Chinese version was developed from the Thai version by the developers. [46] It was tested among 39 Chinese participants between 19 and 29 years old before its use with a Cronbach’s alpha of 0.925. In this sample, Cronbach’s alpha was 0.894. 

#### 2.1.5. Experience in Close Relationships Revised (ECR-R)

ECR-R is an adult attachment measure assessing attachment anxiety and avoidance [47]. The Chinese version demonstrates adequate internal consistency, test–retest reliability and validity (construct and criterion-related) [48]. The 18-item version was used in this study [49]. In this sample, Cronbach’s alpha was 0.875. 

#### 2.1.6. Patient Health Questionnaire (PHQ-9)

The PHQ-9 is a screening tool for depression [50]. The Chinese version of PHQ-9 showed good reliability and validity, and Cronbach’s coefficient was 0.938 [51]. In this sample, Cronbach’s alpha was 0.871.

#### 2.1.7. Rosenberg Self-Esteem Scale (RSES)

The RSES measures global self-worth by both positive and negative feelings about the self [52]. The Chinese version revealed good discrimination indices of items among college students, the consistency reliability coefficient of the scale was 0.8 to 0.89, and the test–retest reliability coefficient was 0.76 [53]. In this sample, Cronbach’s alpha was 0.924.

### 2.2. Statistical Analyses

The prevalence of the BPD symptoms as well as demographic and socio-economic data characteristics were analyzed using frequency, percentage and odds ratio. In addition, Pearson’s correlation coefficient was used to analyze the association between the scores of SI-Bord and MLQ, PSS-10, ECR-R, RSES, RI-9 and PHQ-9. A *p*-value of less than 0.05 was considered significant. Data were analyzed using IBM SPSS 22 (SPSS, Chicago, IL, USA).

## 3. Results

### 3.1. Sociodemographic Characteristics of Participants and Borderline Personality Symptoms by Variables

Table 1 shows the participants’ demographic data and characteristics. Among 767 participants, the mean age was 20.33 ± 1.495 years, and the majority were males (53.5%). A total of 392 (51.2%) participants identified that they got their personal income from parents, 447 (58.8%) perceived their family as middle class. No difference of all variables was observed between BPD and non-BPD groups.

### 3.2. The Prevalence of BPD Symptoms

Participants scoring >7 on the SI-Bord totaled 134 (17.5%). The prevalence did not differ by sex, personal income and perceived familial wealth. 

### 3.3. Pearson’s Correlation between BPD Symptoms and Mental Health Factors

Pearson’s correlation showed that all variables had significant correlations with BPD symptoms. Attachment anxiety, attachment avoidance, depression and perceived stress were positively correlated with BPD symptoms while MIL, resilience and self-esteem were negatively correlated (Table 2).

## 4. Discussion

We identified a significant prevalence of BPD symptoms in this sample, i.e., 17.5%, according to the SI-Bord’s cut-off score >7. The prevalence was in the range of related studies conducted among Chinese students (2.21 to 17.7%) [8,9,10,11,12,13,14] despite using a different tool. Worldwide, variations exist in the reported prevalence of BPD symptoms among university students. A systematic review of 39 studies estimated a lifetime prevalence of 9.7%, ranging between 0.5% and 32.1% [2]. The primary reason for variation in prevalence rate is the instruments used in each study. Conceivably, the study where diagnostic interviews, e.g., PDI-IV, SCID-II, were used, yielded a lower prevalence rate. The results from the current study are best compared with the study among the Thai students as an equal measurement; therefore, the SI-Bord was used. Both studies yielded a similar prevalence when the same cut-off was applied [28]. Interestingly, the prevalence of BPD varied when used outside of Asian culture. 

What might have influenced the prevalence rate is that the study was conducted during the COVID-19 pandemic. The stress during such conditions may have aggravated the BPD symptoms, e.g., the feeling of being abandoned during lockdown, resulting in a (falsely) high stress rate. The findings of this study were consistent with several studies showing the impact of COVID-19 on the mental health of university students [54]. A meta-analysis showed that the prevalence of depression among Chinese university students during the COVID-19 pandemic was 27%. However, students residing in higher-risk areas presented severe anxiety and depression, especially during the late period of the COVID-19 pandemic, when the level of anxiety and depression increased to 42 and 44%, respectively, during the diffusion period attenuation [55]. Another meta-analytic study showed that the general population feared contagion and infecting close contacts, loneliness and boredom associated with quarantine, as well as insomnia, which for college students presented higher rates of anxiety, depression, sleep problems and suicidal ideation [56].

In terms of sex differences, a related survey among a large number of university students in China demonstrated a higher prevalence among females than males [57], and a recent publication among American students found sex differences between females and males [7]. Our findings showed that the prevalence of BPD symptoms among both sexes was equal, which was congruent with those from a related meta-analysis. [2] Studies regarding perceived family wealth or economic status differences among university students with BPD symptoms remain limited. Nonetheless, a related publication before the pandemic in China reported that students from low income families had a higher probability of having BPD than those from average or wealthy families [57]. We found equal socioeconomic status in both groups because this constituted an online study. Internet use was found to be related to higher socioeconomic status [58].

Consistent with the related studies, an insecure attachment style has been linked to BPD symptoms [16,59,60], and especially attachment anxiety [59,61]. The fact that both attachment anxiety and attachment avoidance were correlated with BPD symptoms indicated that BPD was associated with fearful attachment, according to Bartholomew’s model [62,63].

As expected, BPD symptoms were significantly associated with adverse mental health variables such as depression and perceived stress. This was consistent with related studies indicating that patients with BPD commonly co-occurred with depressive symptoms. Up to 80% of patients with BPD experienced one or more episodes of major depressive disorder (MDD) in their lifetime, and up to one half of patients with BPD experienced ongoing MDD. Deficits in emotional functioning have been associated with heightened symptoms across numerous psychological disorders, including depression and BPD [64,65,66]. Altered stress reactivity and self-perception might reflect the emotional inability and lack of insight of adolescents with BPD. Patients with BPD, especially young individuals, are prone to presenting high stress levels and stress episodes which may lead to specific impulsive and harmful behaviors, e.g., self-harm or suicidal attempts, in this population [1].

The same is true for positive mental health variables such as self-esteem, resilience and MIL that were negatively correlated with BPD symptoms. These findings are in line with the theoretical underpinning of BPD that reveals considerable difficulty in maintaining a stable sense of who they believe to be while experiencing dramatic shifts in feelings of self-worth. Even though their evaluation of themselves may not be unambiguously negative, their subjective experience of positive self-states could negatively impact their self-esteem [24]. Patients with BPD feature the social communication problem of inflexibility in the capacity for social communication as well as problems with recalibrating the mind in the face of adverse experiences, in particular when interacting with others, and poor appraisal capacity of a situation, which is opposite the nature of resilience that helps to positively appraise the situation and protect people from harsh conditions [25]. Related studies show that BPD patients present a greater sense of emptiness, low sense of self and emotional dysregulation, resulting in low meaning in life, which may lead to an intolerable experience of self and wish to use self-harm or suicidal behavior to escape their reported pain [26].

### 4.1. Strengths and Limitations

To the best of our knowledge, our study constitutes one of the early studies conducted on the prevalence of BPD among Chinese university students during the COVID-19 pandemic. Furthermore, it provides data for understanding the importance of BPD symptoms and its correlated factors and assists in planning and implementing further prevention and control programs. However, the results of this study should be interpreted with caution due to several method limitations. First, the study used self-reported questionnaires, especially for borderline personality disorder. Therefore, recall bias and social desirability bias are inevitable. Second, the data in this study were obtained from participants with access to computers and smartphones. This procedure may influence the actual prevalence of BPD. Finally, a larger sample size that could reach out to representing students is needed to assess the prevalence of BPD. Further studies on how resilience and meaning in life contribute to depression among students with BPD symptoms should be conducted. 

### 4.2. Practical Implementation

It would be beneficial for educational authorities and universities to offer early screenings for students with a high risk of borderline personality disorder. In addition, universities and instructors should pay attention to both high- and low-risk groups. Schools should have a clear plan to train therapists in counselling techniques or interventions to promote a sense of meaning in life, self-esteem and resilience as well as to reduce the depression and stress experienced by university students. Universities should have a policy to promote and support extracurricular activities among students. In addition, students should be aware of and learn skills to handle BPD symptoms and prevent stigma.

## 5. Conclusions

The prevalence of borderline personality disorder, albeit using different measurements, remains high among Chinese university students. The correlation between BPD symptoms and different mental health factors suggests clues to help university students as well as potential further research on fostering positive mental health variables, such as self-esteem, meaning in life and resilience, to prevent university students from experiencing mental health difficulties such as stress and depression. 

## Figures and Tables

**Table 1:** Characteristics of the participants (*n* = 767).

Variables	Whole	BPD (*n* = 134)	Non-BPD (*n* = 609)	Test Difference	*p*-Value
Gender				Χ^2^(1) = 2.586	0.125
Male	407(53.5)	62(46.3)	328(53.9)		
Female	354(46.5)	71(53.0)	276(45.3)		
Unrevealed	6(0.8)				
Age (years), min–max		18–23	18–25	*t*(741) = 0.332	0.740
Mean ± SD	20.33 ± 1.495	20.38 ± 1.48	20.33 ± 1.50		
Personal income				Χ^2^ (3) = 3.250	0.355
Daily pocket money from parents	392(51.2)	66(17.4)	314(51.6)		
Part-time job	21(2.7)	1(0.7)	20(3.3)		
Both	347(45.3)	66(49.3)	269(44.2)		
Others	6(0.8)	1(0.7)	5(0.8)		
Perceived Family Wealth				Χ^2^(2) = 0.383	0.826
Wealthy	24(3.2)	5(3.7)	18(3.0)		
Middle class	447(58.8)	76(56.7)	356(58.5)		
Poor	289(38.0)	53(18.9)	228(37.4)		
Unrevealed	7(0.9)	-	7(1.1)		

**Table 2:** Correlations between SI-Bord and attachment, meaning in life, perceived stress, resilience, depression and self-esteem.

	SI-Bord (*n* = 767)	Attachment Anxiety (*n* = 765)	Attachment Avoidance (*n* = 761)	MIL (*n* = 767)	Perceived Stress (*n* = 764)	Resilience (*n* = 739)	Depression (*n* = 741)	Self-Esteem (*n* = 742)
SI-Bord	1	0.473 **	0.180 **	−0.148 **	0.481 **	−0.238 **	0.451 **	−0.388 **
Attachment anxious		1	0.171 **	−0.176 **	0.403 **	−0.192 **	0.412 **	−0.360 **
Attachment Avoidant			1	−0.314 **	0.334 **	−0.430 **	0.243 **	−0.323 **
MIL				1	−0.207 **	0.569 **	−0.326 **	0.421 **
Perceived Stress					1	−0.466 **	0.594 **	−0.475 **
Resilience						1	−0.356 **	0.436 **
Depression							1	−0.516 **
Self-Esteem								1

** *p* < 0.001; SI-Bord = Screening Inventory for Borderline Personality Disorder; MIL = Meaning in Life.

## Data Availability

The dataset is available from the corresponding authors upon reasonable request.
